# Phytobioactive compounds as therapeutic agents for human diseases: A review

**DOI:** 10.1002/fsn3.3308

**Published:** 2023-04-17

**Authors:** Muhammad Riaz, Ramsha Khalid, Muhammad Afzal, Fozia Anjum, Hina Fatima, Saadiya Zia, Ghulam Rasool, Chukwuebuka Egbuna, Andrew G. Mtewa, Chukwuemelie Zedech Uche, Muhammad Aamir Aslam

**Affiliations:** ^1^ Department of Allied Health Sciences University of Sargodha Sargodha Pakistan; ^2^ Department of Biochemistry University of Agriculture Faisalabad Pakistan; ^3^ Hill Fruit Research Station Sunny Bank Murree Pakistan; ^4^ Department of Chemistry Government College University Faisalabad Pakistan; ^5^ Department of Basic and Applied Chemistry, Faculty of Science and Technology University of Central Punjab Lahore Pakistan; ^6^ Africa Centre of Excellence in Public Health and Toxicological Research (ACE‐PUTOR), Nutritional Biochemistry and Toxicology Unit University of Port‐Harcourt Port Harcourt Nigeria; ^7^ Chemistry Section, Malawi Institute of Technology Malawi University of Science and Technology Limbe Malawi; ^8^ Department of Medical Biochemistry and Molecular Biology, Faculty of Basic Medical Sciences University of Nigeria Enugu Nigeria; ^9^ Insitute of Microbiology University of Agriculture Faisalabad Pakistan

**Keywords:** alkaloids, medicinal plants, phytobioactive compounds, polyphenols, terpenoids

## Abstract

Phytobioactive compounds are plant secondary metabolites and bioactive compounds abundantly present in medicinal plants and have remarkable therapeutic potential. Oxidative stress and antibiotic resistance are major causes of present‐day ailments such as diabetes, atherosclerosis, cardiovascular disorders, cancer, and inflammation. The data for this review were collected from Google Scholar, PubMed, Directory of Open Access Journals (DOAJ), and Science Direct by using keywords: “Medicinal plants, Phytobioactive compounds, Polyphenols, Alkaloids, Carotenoids etc.” Several studies have reported the pharmacological and therapeutic potential of the phytobioactives. Polyphenols, alkaloids, terpenes, and polysaccharides isolated from medicinal plants showed remarkable antioxidant, anticancer, cytotoxic, anti‐inflammatory, cardioprotective, hepatoprotective, immunomodulatory, neuroprotective, and antidiabetic activities. This literature review was planned to provide comprehensive insight into the biopharmacological and therapeutic potential of phytobioactive compounds. The techniques used for the extraction and isolation of phytobioactive compounds, and bioassays required for their biological activities such as antioxidant, antimicrobial, anti‐inflammatory, and cytotoxic activities, have been discussed. Characterization techniques for the structural elucidation of phytobioactive compounds such as HPLC, TLC, FTIR, GC–MS/MS, and NMR have also been discussed. This review concludes that phytobioactive compounds may be used as potential alternative to synthetic compounds as therapeutic agents for the treatment of various diseases.

## Graphical abstract

Phytobioactive compounds are plant secondary metabolites and have remarkable therapeutic potential. Polyphenols, alkaloids, terpenes, and polysaccharides isolated from medicinal plants showed remarkable antioxidant, anticancer, cytotoxic, anti‐inflammatory, cardioprotective, hepatoprotective, immunomodulatory, neuroprotective, and antidiabetic activities. Phytobioactive compounds may be used as a potential alternative to synthetic compounds and as therapeutic agents for the treatment of various diseases.

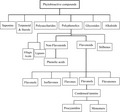

## 1 INTRODUCTION

According to the Worlds Health Organization (WHO), any plants that have therapeutic potential or which are precursors of pharmaceutical drugs are referred to as medicinal plants (Munir et al., 2014). Since the emergence of life, medicinal agents have been used for the treatment of ailments (Shahid et al., 2016). Most of the traditional medicines are obtained from plants (Ashraf et al., 2015). In spite of huge progress in pharmaceutical industry, plants are major raw materials for synthetic drugs (Shang et al., 2021). Clinical advancements have increased the value of medicinal plants by determining active principles present in them (Noreen, Hussain, & Shahid, 2020). Nowadays, the world is progressively turning toward effective herbal medicines (Aanouz et al., 2021). Synthetic drugs have not only side effects and are inadequate but are also expensive and unaffordable in developing nations (Ashraf et al., 2016).

In the last few decades, natural therapeutic compounds are explored by scientists from all across the world (Jilani et al., 2016). Ethnomedicinal survey of plants sets the basis for discovering new medicinal compounds (Khalid et al., 2016). With the passage of time, a large amount of evidence has been gathered that demonstrates the use of plants in the field of pharmacology (Radha et al., 2021). Since the appearance of life on Earth, plants are a major source of food, clothing, shelter, and medicine (Shahzadi et al., 2018; Yousaf et al., 2017). Due to their nutritive and medicinal potential, plants have been playing a vital role in human life (Yazarlu et al., 2021). From the start of life, human civilization has been using plants, mainly as medicines, and most civilizations still use them (Bano et al., 2017). Currently, 25% of pharmaceutical drugs are derived from plants (Okpuzor et al., 2021). In developing countries, about 70%–95% population uses medicinal plants to treat health issues (Liaqat et al., 2019).

As stated by WHO, 80% of the world population uses phytochemical agents for the treatment of diseases. Bioactive compounds of plants have remarkable potential to treat diseases (Hussain et al., 2018). Secondary metabolites, such as phenolics, flavonoids, alkaloids, saponins, and terpenoids produced by plants, are the constituents of plant's defense system but they have remarkable potential to treat various diseases (Aanouz et al., 2021; Anjum et al., 2020). Plant extracts have been gaining huge attention to control emerging antibacterial resistance (Abbas et al., 2020; Safdar et al., 2021). Crude plant extracts have remarkable antibacterial potential (Romano et al., 2021). Purified plant bioactive compounds are a major research topic among researchers (Lim et al., 2021; Shahid et al., 2021).

Phytobioactive compounds play a significant role in the adaptation of plants to their surrounding environment but are also a major source for the pharmaceutical industry (Merrouni & Elachouri, 2021). Phytobioactives are also known as plant secondary metabolites and bioactive compounds (Fatima et al., 2021). They are present ubiquitously in the plant kingdom and are considered nonnutritional but vital components for the maintenance of human health (Nafees et al., 2017). Phytobioactive compounds are not required for the basic metabolism of plants, are synthesized apart from the primary biosynthetic pathway, and are synthesized from the metabolites of biosynthetic routes (Riaz et al., 2017). Several of them not only play important roles for the plants such as protecting, attracting, signaling, and adapting to their environment but also represent the main source of pharmaceuticals (Jahan & Shahid, 2019; Khairan et al., 2021). The foundation of modern therapeutics is based on the use of plants and their extract in preparing herbal drugs, enabling man in establishing an empirical medicinal system (Gumisiriza et al., 2021). Thus, screening of a number of plant species is being carried out for these herbal compounds (Waseem et al., 2021).

## 2 HISTORY

The history of medicinal plants is as old as humankind. In the beginning, people exploited herbs for nutrition but following the discovery of medicinal attributes, medicinal plants became a beneficial source for the improvement of health among human communities (Kumar et al., 2021). Herbal products were introduced by the Romanian pharmacopeia during the 19th century by establishing the first institute of medicinal herbs in Cluj city in 1904 (Vinatoru, 2001). The herbal plants' usage in ancient times truly illustrates the history of bioactive molecules (Swargiary et al., 2021). People were unaware of plants' bioactives but the usage of these molecules is sufficiently diverse in various prospects (Zeidali et al., 2021). Typically, secondary metabolites are the bioactive compounds produced by plants. The use of different parts of plants (bark or flowers, leaves, berries, roots, and seeds) for healthcare in treating various diseases is called herbal medicine (Anwar et al., 2013).

During the late 19th century, researchers began to isolate, purify, and identify phytobioactive compounds from plants, and their efforts led them to discover vital drugs from plants that are the base of modern medicine (Tanweer et al., 2018). In this view, for the preparation of semi‐synthetic drugs, bioactive compounds isolated from medicinal plants have been modified to make them effective. In 1953, aspirin was synthesized through the structural modification of an active constituent of a number of medicinal plants, which is salicylic acid, having pain‐relieving effects (Shahid et al., 2012).

## 3 LITERATURE SEARCH STRATEGY

The data for this review were collected by searching databases including google scholar, PubMed, Science direct, Directory of Open Access Journals (DOAJ), Scopus, and Web of Science by using keywords: “Medicinal plants, Therapeutic potential of plants derived Bioactive compounds, Phytobioactive compounds, phytochemicals, phytoconstituents, Polyphenols, Flavonoids, Alkaloids, Terpenoids, Carotenoids, Gums etc.” Research articles and reviews published in English were only considered for this review study. Publications that extensively investigated the biological activities and therapeutic effects of bioactive compounds present in plants were included. Moreover, the published research conducted on animal models for in vitro and in vivo experimentations were also included.

## 4 PLANT‐DERIVED BIOACTIVE COMPOUNDS AND THEIR THERAPEUTIC POTENTIAL

**FIGURE 1:**
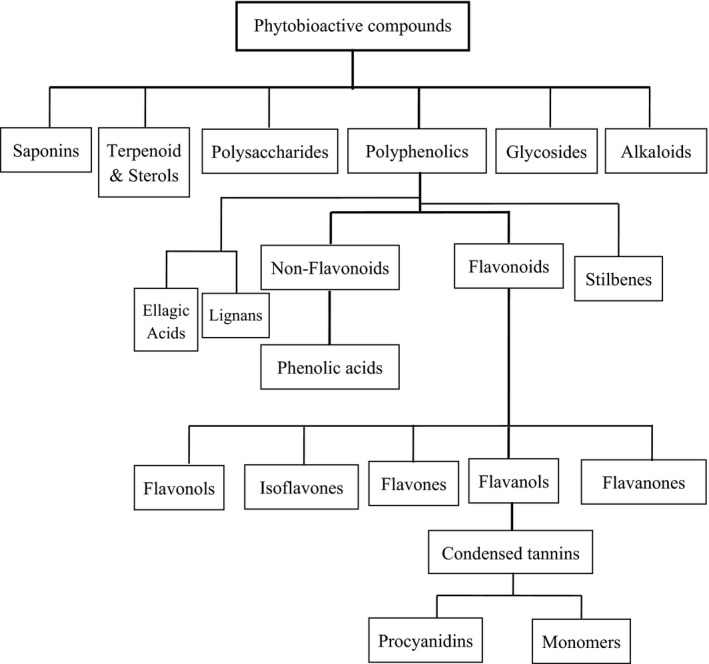
Classification of phytobioactive compounds.

**FIGURE 2:**
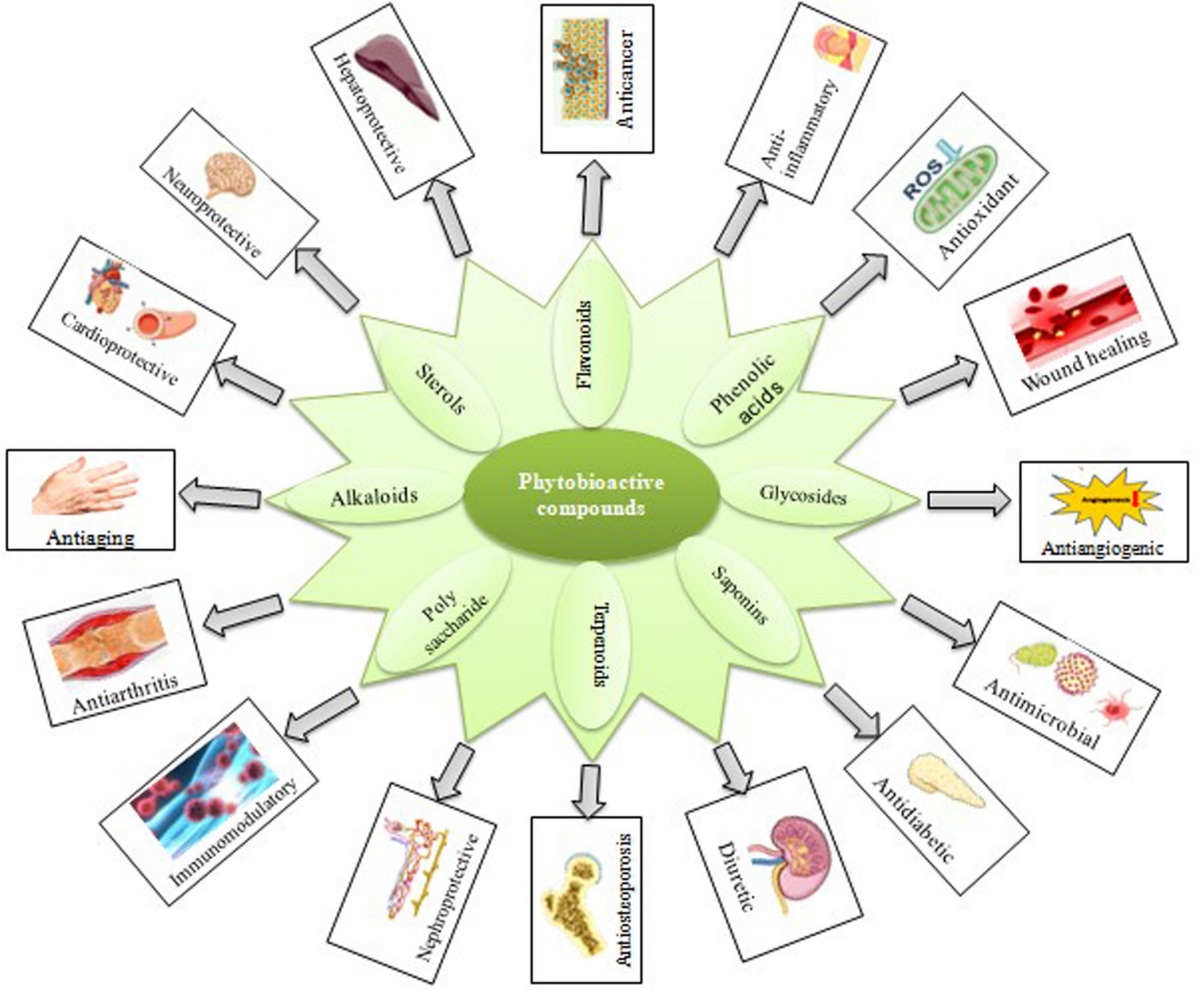
Overview of the therapeutic potential of phytobioactive compounds.

**FIGURE 3:**
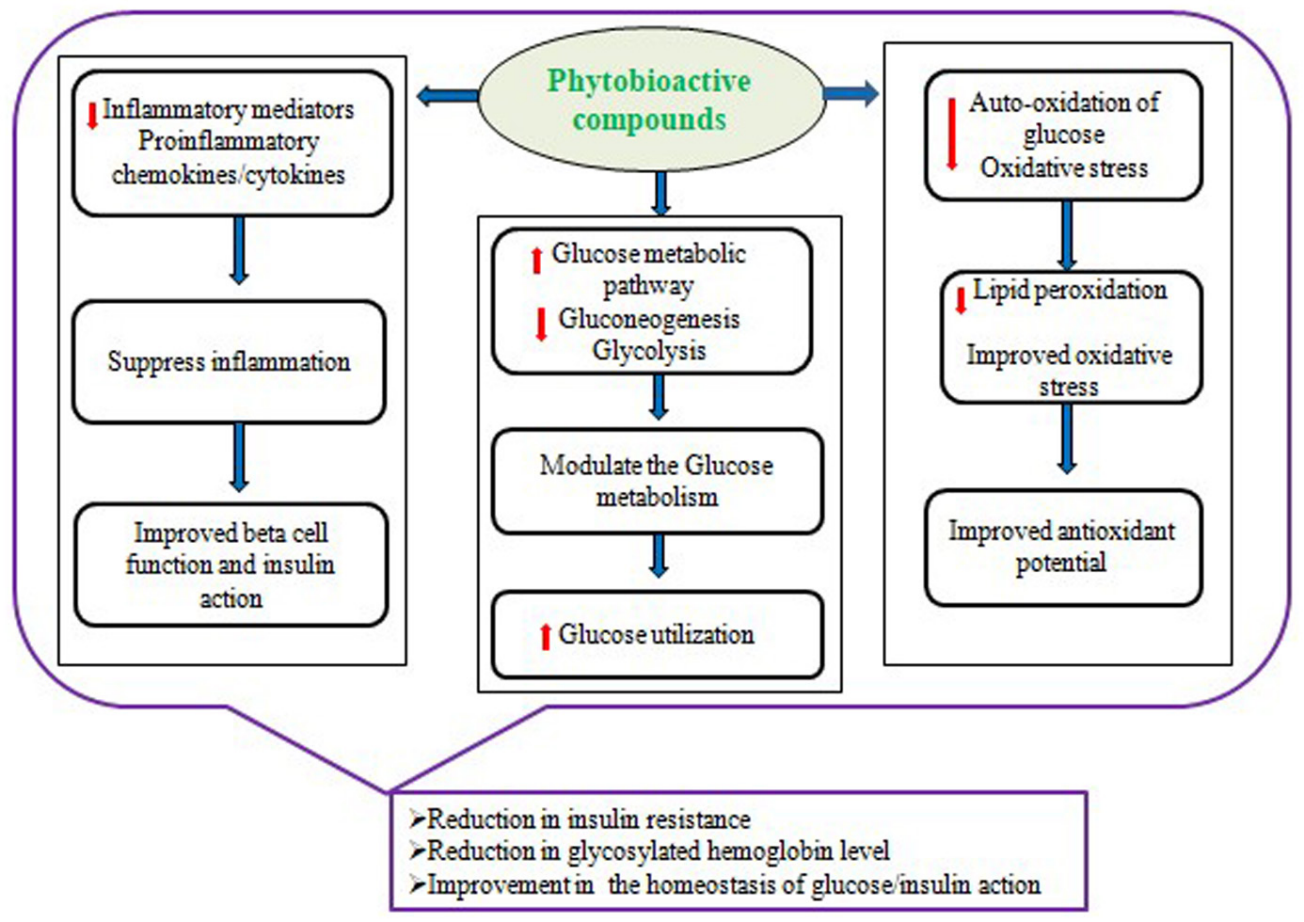
Schematic mechanism of phytobioactive compounds in treating type 2 diabetes.

Phytobioactive compounds are the compounds present in plants that provide protection against various diseases but are not of dietary importance. These compounds obtained from plants having distinct structural and functional properties are referred to as phytochemicals or secondary metabolites of plants. These are found abundantly in vegetables, grains, fruits, seeds, and nuts. These metabolites are usually classified into six large‐molecule families: phenolics, terpenes, alkaloids, saponins, glycosides, and polysaccharides based on their biosynthetic pathways. The schematic classification of phytobioactive compounds is shown in Figure 1, whereas the overview of the therapeutic potential of phytobioactive compounds is presented in Figure 2. Phytobioactive compounds have been extensively used in traditional medicines for the treatment of various diseases including type 2 diabetes. Higher antidiabetic potential of these phytobioactives has been shown in various animal models, and plant bioactive‐based medicines currently are in great demand in the market due to their multiple efficacies and higher availability. The possible mechanism of molecular action of phytobioactives for the treatment of type 2 diabetes is shown in Figure 3. There are many complications associated with type 2 diabetes, and few of the regularly used phytobioactive compounds through food have shown antidiabetic potential in a variety of ways including the reduction in drug loads during treatment (Ganesan et al., 2017). A summary of the biological/pharmacological properties of phytobioactive compounds and their action mechanism is given in Table 1.

**TABLE 1:** Biological/pharmacological properties of phytobioactive compounds and their mechanism of action.

Major class of bioactive compounds	Subclasses	Examples	Sources/plants	Biopharmacological activities	Action mechanism	References
Phenolic acids	Hydroxybenzoic acid	Gallic acids 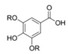	Gallnuts, witch hazel, sumac, tea leaves, bark, oak, and other plants	Induction of antiproliferation and apoptosis	Regulation of mitochondrial‐mediated pathways, induction of the activity of ROS, caspase‐3, and caspase‐9, reduction of the mitochondrial membrane potential, and the elevation of Bcl‐2‐like protein 4	Jagan et al. (2008)
				Induce HSC apoptosis	Regulating TNF‐α signaling pathway	Chang et al. (2015)
		Ellagic acid 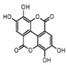	Berries, grapes, pomegranates, green tea, wine, walnuts, and chocolate	Anticarcinogenesis	Removal of free radicals and prevention of DNA fragmentation	Hussein and Khalifa (2014)
				Prevention of inflammasome‐associated PAH	Inhibition of NLRP3 inflammasome activation	Tang et al. (2015)
	Hydroxycinnamic acid	Chlorogenic acid 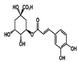	*Hibiscus Sabdariffa* leaves, peaches, and eggplants	Inhibition of HepG2 xenograft proliferation and progression	ERK1/2 inactivation and suppression of MMP‐2 and MMP‐9 expression	Yan et al. (2017)
				Inhibition of proliferation and profibrogenesis‐related genes in HSCs	Improvement of antioxidant capacity via Nrf2 pathway activation and suppression of profibrotic action through NOX/ROS/MAPK pathway inhibition	Shi et al. (2016)
				Attenuation of antifibrosis and liver injury	Suppression of the AGEs‐mediated induction of RAGE gene expression by stimulating GSH and increasing PPARγ	Stefanska (2012)
		Curcumin 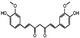	*Curcuma longa*	Antioxidant, anticarcinogenic, and anti‐inflammatory	Decrease oxidative damage and inhibit apoptosis in myocardium (JAK2/STAT3 signal pathway)	Sultana et al. (2021)
		6‐Gingerol 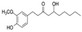	*Curcuma longa*	Antioxidative, anti‐inflammatory, and antiapoptotic	Alleviate oxidative stress to inhibit cell death (upregulate PI3K/Akt signaling pathway expression)	Zhang et al. (2017)
Flavonoids	Anthocyanins	Delphinidin 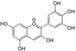	*Ribes nigrum* L., bilberry and blueberry	Inhibit tumor growth, antiproliferation, apoptosis, and antiangiogenesis	Activation of caspase‐3/9, suppression of Bax, Akt/PI3K/MAPK, VEGFA, cyclin D1, PCNA, Bcl‐2, and EGFR	Pal et al. (2013)
				Antiangiogenesis	Suppress Akt/mTOR/PI3K/p70S6K, ERK, HIF‐1α, CoCl2, EGF, and VEGF mRNA	Kim et al. (2017)
		Cyanidin 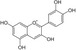	*Ipomoea batatas*, *Morus alba* L.	Antioxidant, antifibrotic and anti‐inflammatory	Attenuate inflammation of the liver cells through the inhibition of NLRP3 inflammasome activation, lowering the cleaved IL‐1β	Wang et al. (2017)
		Malvidin 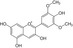	*Ribes nigrum* L.	Anti‐inflammatory	Increase the cytosolic IκB which is an NF‐κB inhibitor and inhibit nuclear translocation of NF‐κB	Bishayee et al. (2013)
	Flavanols	EGCG 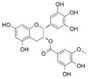	Tea	Inhibit metastasis	Decrease the half‐life of osteopontin mRNA by reducing osteopontin	Zapf et al. (2015)
				Anticarcinogenesis	Regulate the self‐renewal Hh/Gli1, Wnt/beta‐catenin pathways, and related genes EGFR, cMyc, and cyclin D1 along with E‐cadherin downregulation	Sur et al. (2016)
				Induce apoptosis and antiproliferation	Receptor tyrosine kinase inhibition; PI3K/AKT downregulating; and inactivation of NF‐κB via downregulation of Bcl‐2 alpha and Bcl‐xl	Shimizu et al. (2008)
	Flavanones	Hesperidin 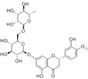	Citrus fruits	Inhibition of metastasis	Reducing the expression of MMP‐9 via inhibiting the activated AP‐1 and NF‐κB activity by p38, JNK, and I κB signaling pathways	Yeh et al. (2009)
				Induce apoptosis	Regulation of mitochondrial pathway and death receptor pathway; trigger the mitochondrial pathway activation by increasing the intracellular ROS, ATP, and Ca^2+^ levels	Banjerdpongchai et al. (2016), Zhang et al. (2015)
		Naringenin 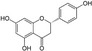	Citrus fruits	Inhibition of invasion and metastasis	Suppress MMP‐9 transcription through the inhibition of NF‐κB and AP‐1 activity	Yen et al. (2015)
	
Eriodictyol 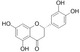	*Eriodictyon californicum*	Induction of apoptosis	Upregulating PARP and Bax, and downregulating the Bcl‐2 protein	Wang et al. (2016)
	Flavones	Apigenin 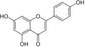	*Scutellaria baicalensis* Georgi	Inhibition of HSC Activation	Dependent on mitochondrial‐activated Apoptosis	Akram, Riaz, Wadood, et al. (2020), Jing Li et al. (2011)
		Chrysin 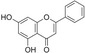	*Passiflora caerulea*, the passion flowers, propolis, and honey	Induce apoptosis, decrease the proliferation and cell motility	Downregulate the expression of Skp2 and LRP6; activate p53/Bcl‐2/caspase‐9 pathway	Huang et al. (2016)
				Inhibit proliferation and profibrogenesis‐related genes in HSCs	Suppressing TGF‐β1/Smad pathway	Balta et al. (2015)
		Luteolin 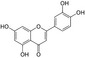	Green pepper, celery, dandelion, thyme, and parsley	Induction of apoptosis	Regulation of extrinsic and intrinsic caspases along with executioner caspases through ROS‐mediated pathway	Chen et al. (2021)
				Inhibition of proliferation‐ and profibrogenesis‐related genes in HSCs	Suppress the phosphorylation of AKT and Smad pathway simulated by PDGF and TGF1; Induce G1 arrest with reduced expression of cyclin E, bcl‐2, and p‐Cdk‐2; Increase p53 expression and caspase 3 activity	Li et al. (2015)
	Flavonols	Quercetin 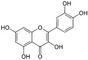	Berries, tea, beans, broccoli, and apples	Inhibit carcinogenesis	Upregulate BAX and p53 via downregulating ROS, COX‐2, PI3K, and PKC	Maurya and Vinayak (2015)
				Inhibition of HSC activation	MMPs activation and the regulation of antifibrogenic/ profibrogenic molecules balance	Hernández‐Ortega et al. (2012)
		Kaempferol 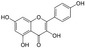	Rosa damascene, delphinium, grapefruit	Autophagy‐mediated cell death	ER stress–CHOP–autophagy signaling pathway	Akram, Riaz, Munir, et al. (2020), Chen et al. (2017)
		Myricetin 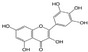	Red wine, tea, berries, nuts, fruits, and vegetables	Inhibition of HCC development	Inhibit PAK1 via coordinate abrogation of PI3K/AKT and MAPK/ERK	Iyer et al. (2015)

	Isoflavonoids	Genistein 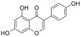	Soy	Inhibition of metastasis	Reverse the epithelial–mesenchymal transition, partly mediated through nuclear factor of activated T cell 1	Chen et al. (2021)
Puerarin 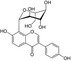		Root of Pueraria	Induce apoptosis	Regulating MAPK pathway	Zhang et al. (2017)
Lignans	–	Sauchinone 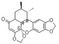	*Schisandra chinensis*	Antioxidant and anti‐inflammatory	Inhibit p38 phosphorylation and signaling pathways of JNK death	Kim et al. (2012)
		Isovaleroylbinankadsurin A 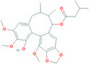	*Schisandra Chinensis*	Antioxidative, antiapoptotic and anti‐inflammatory	Inhibit ROS generation and blocked the apoptosis (activating RISK pathway dependent on GR)	Zuo et al. (2020)
Glycosides	Stilbenes	Resveratrol 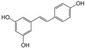	Grapes, berries, and red wine	Antiangiogenesis	Inhibit the expression of VEGF via NF‐κB‐mediated mechanism	Yu et al. (2010)
				Inhibit profibrogenesis‐ and proliferation‐related genes in HSCs	Suppress NF‐κB and Akt activation	Zhang et al. (2016)
				Inhibit the activation of HSC	Dependent on mitochondrial‐activated apoptosis	Li et al. (2011)
				Inhibit invasion and metastasis	Regulate HGF‐c‐Met signaling pathway; reduce MMP‐9 via downregulating NF‐κB signaling pathway	Gao et al. (2017)
		Polydatin 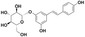	*Reynoutria japonica* Houtt	Antioxidant and anti‐inflammatory	Promote autophagic flux clearing injured mitochondria reducing ROS and cell death	Ling et al. (2016)
Terpenes	Triterpenoid	Asiatic acid 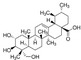	*Centella asiatica*	Anticancer activity	Inhibit cell proliferation, migration, and angiogenesis (inhibition of Src/FAK/ERK and PI3K/Akt signaling pathway).	Mioc et al. (2022)
				Neuroprotective activity	Downregulate IL‐6, IL‐1β, and TNF‐α; downregulate Bax and upregulate Bcl (NF‐kB/STAT3/ERK signaling pathway inhibition). Antiapoptotic and anti‐inflammatory effects (inhibit TAK1–JNK pathway).	Lu et al. (2019), Mioc et al. (2022)
				Cardioprotective activity	Antioxidant effect (inhibit MAPK/mitochondria‐dependent apoptotic pathway); reduces myocardial hypertrophy (activation of Akt/GSK‐3β‐mir‐126‐mediated signaling pathway).	Yi et al. (2022)
				Hepatoprotective activity	Suppression of PERK/ATF6 and IRE1 pathway activation involved in the initiation of apoptosis. Regulatory factor on the Bcl‐2/Bax and PI3K/AKT/mTOR signaling pathways preventing liver fibrosis	Wang, Lao, et al. (2018), Wei et al. (2018)
				Anti‐inflammatory activity	Inhibits NF‐κB pathway activation and the nucleotide‐binding domain‐like receptor protein 3 (NLRP3) inflammasome, which mediates IL‐1β release and maturation	Yuyun et al. (2018)
	
		Oleanolic acid 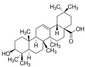	*Ligustrum lucidum*, *Olea europaea*, *S. mussotii*, and Olives	Anticancer activity	Inhibit cell migration (upregulation of PI3K/Akt pathway; inhibit cell proliferation; inhibition of notch signaling pathway); anti‐inflammatory activity (suppress NF‐kB signaling pathway); and induce apoptosis (activation ERK/JNK/p38 pathway)	Mioc et al. (2022)
				Antidiabetic activity	Reduces inflammatory cytokines TNF‐α and IL‐6 (NF‐kB signaling inhibition); reduces proinflammatory cytokines expression (NLRP3 inflammasome inhibition); and antioxidant effect (inhibition of MAPK signaling)	Mioc et al. (2022), Swanson et al. (2019)
Neuroprotective activity	Prevent microglial migration toward neurons and removes intracellular ROS. Decreased production of ROS and MDA Facilitate the Nrf2 signaling activation and upregulate its downstream stress response protein HO‐1 in the injured cortex			Shi et al. (2021)
	
		Ursolic acid 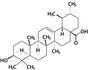	Fruits, vegetables, dietary fibers, and herbs of the Lamiaceae family	Anticancer activity	Inhibit cell proliferation, survival, and angiogenesis (inhibit PI3K/Akt signaling pathway); induce apoptosis (activation of JNK signaling pathway)	Guo et al. (2020)
				Antidiabetic activity	Modulate AGEs‐RAGE signaling pathway, upregulation of MMP‐2 protein levels, and inhibition of proinflammatory cytokines TNF‐α, MCP‐1, and TGF‐β1 production	Dai et al. (2021)
				Anti‐infectious Activity	Stimulate the anti‐inflammatory cytokines GM‐CSF, IL‐10, and IL12 along with the ROS and NO production in parasite‐infected immune cells. Inhibit the expression of proinflammatory cytokines IL‐1β, IL‐6, TNF‐α, and TGF‐β1	Choi and Lee (2019)
Anti‐inflammatory activity	Inhibit PERK activation, resulting in Bax proapoptotic protein downregulation and Bcl‐2 antiapoptotic protein upregulation (inhibit the NF‐kB and MALAT1/miR206/PTGS1 signaling pathway).			Mou et al. (2021)
	Sesquiterpene lactone	Artemisinin 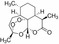	*Artemisia annua* L.	Anti‐inflammatory, antioxidant, and antitumor	Suppress NLRP3 inflammasome activation (cleaved IL‐1β, caspase‐1, decrease NLRP3, and ASC)	Wang et al. (2020)
	Diterpene lactone	Ginkgolide B 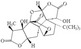	Ginkgo leaves	Anti‐inflammatory, antioxidant, and antiapoptotic	Inhibit apoptosis induced by ER stress via PI3K/AKT/mTOR signaling pathway	Guo et al. (2019)
Alkaloids	–	Berberine 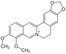	*Berberis vulgaris* L. and *Coptis Chinensis*	Antiapoptotic and anti‐inflammatory	Promote proliferation, attenuate apoptosis through mitophagy‐mediated HIF‐1α/BNIP3 pathway	Zhu et al. (2020)
		Capsaicin 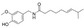	*Capsicum annuum* L.	Antioxidant and antiapoptotic	Attenuate ROS production, inhibit the opening of mPTP and activation of caspase‐3, downregulation of Bax, upregulation of 14–3‐3η and Bcl‐2, and eventually decreased apoptosis	Huang et al. (2018)
		Matrine 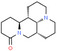	*Sophora flavescens* Aiton	Antitumor and anti‐inflammatory	Upregulates HSP70 expression and activates JAK2/STAT3 pathway	Guo et al. (2018)
Saponins	–	Polyphyllin I 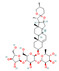	*Paris polyphylla*	Anticancer effect	Inhibit inflammatory responses and oxidative stress (NF‐κB, p65 signaling pathway)	Huang et al. (2020)
		Platycodin D 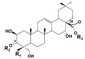	*Platycodon grandiflorus*	Antioxidant, anti‐inflammatory, and antiapoptotic	Inhibit apoptosis and oxidative stress (induce Akt/Nrf2/HO‐1 pathway activation)	Wang, Che, et al. (2018)
		Gypenoside A 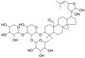	*Gynostemma pentaphyllum*	Anti‐inflammatory, antioxidative, and antitumor	Suppress miR‐143‐3p through AMPK signaling activation	Chang et al. (2020)
		Ginsenoside Rb3 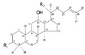	*Panax ginseng* C. A. Meyer	Antiapoptotic	Inhibit apoptosis (activation of JNK/NF‐κB signaling pathway)	Ma et al. (2014)
Polysaccharides	–	Fucoidan 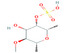	Species of brown algae (Phaeophyta)	Anticancer	Block P‐selectin‐mediated neutrophil rolling on the vessel wall	Omata et al. (1997)
Carotenoids	–	Lycopene 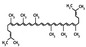	Tomatoes and tomato products, apricots, watermelon, and guava	Antioxidant, anti‐inflammatory, and cardioprotective	Inhibit inflammation and accumulation of ROS (JNK signaling pathway)	Przybylska and Tokarczyk (2022)
Coumarin	–	Osthole 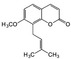	*Angelica pubescens Maxim* and *Cnidium monnieri* (L.) Cusson	Antioxidant, antiapoptotic, and anti‐inflammatory	Exerted anti‐inflammatory and antioxidant effect (inhibit the IκB‐α/NF‐κB signaling pathway and HMGB1 expression)	Wang et al. (2013)

Abbreviations: AGEs: Advanced glycation end products; AMPK: AMP‐activated protein kinase; ATF6: Activating transcription factor 6; BNIP3: BCL2/adenovirus E1B 19 kDa protein‐interacting protein 3; COX‐2: Cyclooxygenase‐2; EGF: Epidermal growth factor; EGFR: Endothelial growth factor receptor; ERK: Extracellular signal‐regulated kinase; ERK: Extracellular signal‐regulated kinase; GM‐CSF: Granulocyte–macrophage colony‐stimulating factor; GSH: Glutathione reductase; HCC: Hepatocellular carcinoma; HGF: Hepatocyte growth factor; HIF‐1α: Hypoxia‐inducible factor 1‐alpha; HMGB1: High‐mobility group box protein 1; HSC: Hepatic stellate cells; HSP70: Heat shock protein 70; IL‐1β: Interleukin 1 β; IL‐6: Interleukin‐6; IRE1: Inositol‐requiring enzyme 1; JAK2: Janus kinase 2; JNK: Jun N‐terminal Kinase; LRP6: LDL receptor‐related protein 6; MAPK: Mitogen‐activated protein kinase; MAPK: Mitogen‐activated protein kinase; MCP‐1: Monocyte chemoattractant protein‐1; MDA: Malondialdehyde; MMP‐2: Matrix metallopeptidase 2; MMP‐9: Matrix metallopeptidase 9; mTOR: mechanistic target of rapamycin; NF‐κB: Nuclear factor kappa B; NLRP3: Nucleotide‐binding oligomerization domain, leucine‐rich repeat‐containing gene family, and pyrin domain‐containing 3; NOX: Nitrogen oxides; Nrf2: Nuclear factor‐erythroid factor 2‐related factor 2; p70S6K: phosphoprotein 70 ribosomal protein S6 kinase; PAH: Pulmonary artery hypertension; PAK1: P21‐activated kinase 1; PARP: poly‐ADP ribose polymerase; PCNA: Proliferating cell nuclear antigen; PERK: Protein kinase‐like endoplasmic reticulum kinase; PI3K: Phosphoinositide 3‐kinase; PKC: Protein kinase C; PPARγ: Peroxisome proliferator‐activated receptor gamma; PTGS1: Prostaglandin‐endoperoxide synthase 1; RAGE: Receptor for advanced glycation end products; RISK pathway: Reperfusion injury salvage kinase pathway; ROS: Reactive oxygen species TNF‐α: Tumor necrosis factor α; STAT3: Signal transducer and activator of transcription 3; TGF‐β1: Transforming growth factor beta 1; VEGF: Vascular endothelial growth factor; VEGFA: Vascular endothelial growth factor A.

### 4.1 Polyphenols

Polyphenols are plants' secondary metabolites or the plants' nonnutritional natural products found in fruits, seeds, and vegetables. Polyphenols are common in the plant kingdom and are a large family of bioactive metabolites derived from secondary metabolism. Most of the polyphenols are derived through phenylpropanoid pathway from l‐phenylalanine. These are characterized by the presence of one or more phenolic groups that show highly diverse structures, of which, flavonoids, phenolic acids, lignans, stilbenes, tannins, and coumarins are the major structural type of polyphenols (Quideau et al., 2011). Some of the polyphenols are responsible for the color, aroma, and antioxidant properties of nuts, vegetables, fruits, and seeds that we consume in our daily life. The importance of polyphenols is increasing particularly because of their health benefits. Their antioxidant role in preventing and treating various diseases is increasing particularly against oxidative stress, cancer, cardiovascular, inflammatory, neurodegenerative, and age‐related degenerative diseases (Hano & Tungmunnithum, 2020). They have a broad range of uses as pharmaceuticals, food supplements, and cosmetic additives. Identification of more than 8000 polyphenols has been carried out from plants, and several hundred have been characterized from grains, vegetables, and fruits (Shen et al., 2017). Various classes of polyphenols are shown in Figure 1 and are described below.

**FIGURE 4:**
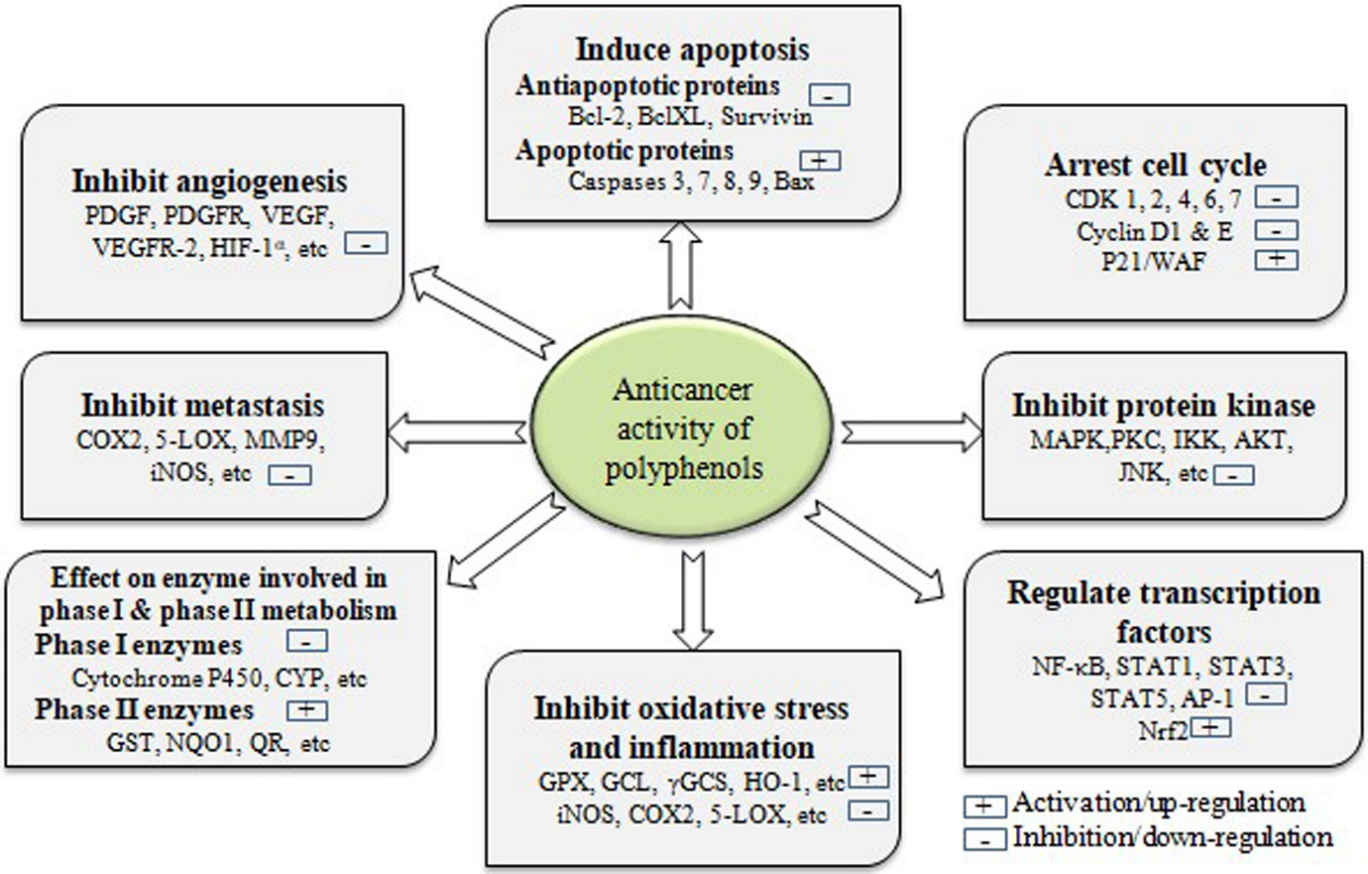
Molecular mechanism of the anticancer activity of polyphenolic compounds. 5‐LOX, lipoxygenase; AP‐1, activator protein‐1; CDK, cyclin‐dependent kinase; COX2, cyclooxygenase 2; CYP, cytochrome P; GCL, glutamate cysteine ligase; GPX, Glutathione peroxidase; GST, glutathione S‐transferase; HIF‐1 α, hypoxia‐inducible factor 1α; HO‐1, hemeoxygenase‐1; IKK, IκB kinase; iNOS, inducible nitric oxide synthase; JNK, c‐Jun N‐terminal Kinase; MAPK, mitogen‐activated protein kinases; MMP9, matrix metalloprotease‐9; NF‐ κB, nuclear factor‐κB; NQO1, nicotinamide adenine dinucleotide phosphate, quinone oxidoreductase; Nrf2, NF‐E2‐related factor 2; PDGF, platelet‐derived growth factor; PDGFR, PDGF receptor; PKC, protein kinase C; QR, quinone reductase; VEGF, vascular endothelial growth factor; VEGFR, VEGF receptor; γGCS, γ‐glutamylcysteine synthetase.

Polyphenols play an important role to prevent carcinogenesis leading to the decreased incidence of some cancers. They have widespread medicinal applications with reduced toxicity and hence, can be used as chemopreventive agents. Any of the endogenous or exogenous carcinogens, tumor promoters, and inflammatory cytokines can take part in the activation of carcinogenesis through the regulation of transcription factors, AP‐1 and NF‐kB; proapoptotic proteins, PARP and caspases; the protein kinases, MAPK, c‐Jun N‐terminal kinase (JNK), and IκB kinase (IKK); growth factor signaling pathways; and the cell cycle proteins, CDK and cyclins (Shen et al., 2017). During the initiation, carcinogens cause damage by interacting with cellular DNA, therefore, blocking the damage caused by genotoxic chemicals can be an effective approach for the prevention of cancer that might be achieved through the elimination of ROS and induction of phase II conjugating enzymes. In the next stage, inhibition of cell proliferation is a useful strategy, such as through the modulation of apoptosis and cell cycle arrest. In progression step, prevention of malignant cells' progression to metastasis and invasiveness and/or the interruption of angiogenesis are particularly important (Ramos, 2008; Shen et al., 2017). Evidence indicates that polyphenols of dietary importance play a protective role in a multistep process of carcinogenesis through phase I and phase II enzymes, cell proliferation, cell cycle progression, DNA repair, metastasis, angiogenesis, and apoptosis (Shen et al., 2017). The possible action mechanisms through which the carcinogenic processes are inhibited by polyphenols are illustrated in Figure 4.

Polyphenols scavenge the excess free radicals (ROS) inhibiting the oxidative stress through interaction with protein kinase‐like endoplasmic reticulum kinase (PERK) dependent on RNA, phosphatidylinositol 3‐kinase (PI3K), protein kinase C (PKC), and mitogen‐activated protein kinases (MAPKs) as these kinases upregulate Nrf2 through phosphorylation. Polyphenols have the capability to suppress the activation of NF‐κB and inhibit the expression of COX2, interleukin (IL)‐1β, and inducible nitric oxide synthase (iNOS), hence decreasing the adoptive cellular responses (Shen et al., 2017). Polyphenols like curcumin, caffeic acid, quercetin, EGCG, and resveratrol have been reported to inhibit oxidative stress. EGCG enhances the antioxidant capacity of cells through MAPK protein activation. Quercetin increases the antioxidant enzymes expression such as γ‐glutamylcysteine synthetase (γGCS), glutathione peroxidase (GPX), and superoxide dismutase (SOD), upregulates glutathione reductase (GSH) level, enhances the stabilization of Nrf2, and promotes the transcription activity mediated by Nrf2. Quercetin also inhibits the activation of NF‐κB. Resveratrol inhibits oxidative stress through the upregulation of γGCS and hemoxygenase‐1 (HO‐1) expression and increases the Nrf2 transcriptional activity (Csiszar et al., 2011; Shen et al., 2017). EGCG reduces the cyclin D1 expression, induces the expression of p27Kip1 and p21waf1/Cip1 genes, and inhibits CDKs 2 and 4 thereby blocking the progression of cell cycle MCF‐7 at G1 phase. Many polyphenols such as curcumin, ellagic acid, apigenin, resveratrol, EGCG, and quercetin induce apoptosis to inhibit carcinogenesis (Shen et al., 2017). Quercetin, theaflavin, catechin, curcumin, and resveratrol through AP‐1 inhibition can inhibit tumor metastasis. Polyphenols including ellagic acid, genistein, quercetin, resveratrol, curcumin, and ECGC can act as inhibitors of NF‐kB (Aggarwal & Shishodia, 2006).

#### 4.1.1 Phenolic acids

Phenolic acids are characterized by the presence of one or more OH groups linked to benzene or any other aromatic ring. Phenolics are responsible for different color development in the plants, help in pollination, and protect plants from pathogenic attack (Ayub et al., 2017). Vegetables, fruits, wine, coffee, and tea are the richest sources of phenolic compounds. Distribution of phenolic compounds is not uniform in plants. Polyphenols are fundamental in ensuring the reproduction, development, and growth of plant (Den et al., 2019). Phenolics have been classified on the basis of the pattern of their basic skeleton into six different classes which were further subdivided into family in order to hierarchize their differences (Naz et al., 2016). The increasing complexity of basic skeleton of the members characterized the first five classes while the sixth class includes a separate group of compounds called hybrid phenolics (Sharif et al., 2018).

Phenolic compounds have important properties, including antimicrobial activity, stabilization of ascorbic acid, inhibition of lipid peroxidation, and carcinogenesis (Sharif et al., 2018). Flavonoids are found in garlic and are known to be helpful in reducing cholesterol level, coronary thrombosis, atherosclerosis, and a variety of other life‐threatening diseases (Ali et al., 2021). The radical‐scavenging activity of flavonoids is ascribed to their capacity of donating hydrogen. Flavonoids contain the phenolic groups which act as a source of hydrogen atoms readily available (Tanweer et al., 2018). Flavonoids have the potential to inhibit fluids that caused diarrhea by targeting the intestinal cystic fibrosis transmembrane regulator (Ahmad et al., 2021). The therapeutic functions of flavonoids comprise protection against allergies, platelet aggregation, free radicals, inflammation, ulcers, microbes, viruses, and tumors (Abbas et al., 2012). Due to physiological, pharmaceutical, and ecological roles, phenolic compounds have been explored and manipulated extensively (Hussain et al., 2018).

Bioavailability of phenolic compounds is limited due to their less water solubility and instability at low pH values (Malode et al., 2021). The bioavailability of polyphenols can be increased by using nanoparticles (Irshad et al., 2020). One of the flavonols called quercetin belongs to flavonoids ubiquitously present in vegetables and fruits. Several in vitro and in vivo studies have demonstrated the antioxidant and anti‐inflammatory potential of quercetin (Afzal, Shahid, & Jamil, 2014). Quercetin has antioxidant activity due to its potential to scavenge free radicals, metal cation chelating ability, and hydrogen atoms or electrons donating ability (Irshad et al., 2017). Quercetin and its glycosidic metabolites modulate the biological processes due to its antioxidant properties and these processes include the reduction of oxidative deoxyribonucleic acid (DNA) damage and cell signaling pathways (Sohaib et al., 2017).

Onion shells (*Allium cepa*) contain a large amount of quercetin (3, 5, 7, 3′, 4′‐pentahydroxy flavones), a natural dye that imparts yellow‐reddish brown color, exhibiting antioxidant, anticarcinogenic, and good dyeing potential (Rehman et al., 2013). Dietary quercetin can prevent the oxidative deterioration of low‐density lipoproteins (LDL) due to its ability to chelate metal ions produced through lipid oxidation and scavenging of free radicals (Sohaib et al., 2015). Honey showed remarkable antioxidant and antitumor activity (Afzal, Shahid, Iqbal, & Hussain, 2014). The phenolics and flavonoids isolated from honey possessed antioxidant and antitumor properties as immunotherapeutic agents (Noor et al., 2014).

Contrary to this, simple phenols (e.g., catechol, hydroquinone, eugenol, p‐anisaldehyde, and phloroglucinol), the C6‐C3 phenylpropanoids, and their derivatives (myristicin, ferulic acid, cinnamic acid, caffeic acid, and sinapyl alcohol), the C6‐C1 benzoic acids (vanillic acid gallic acid and protocatechuic acid), coumarins (dicoumarol, scopoletin, and warfarin), hydrozable tannis (ellagitannins and gallotannins), lignans, and related compounds all belong to nonflavonoid phenolic compounds (Irshad et al., 2018). Phenolic compounds such as flavonoids are present in all plant parts and have remarkable biological activities due to their redox potential (Khan et al., 2012).

Phenolic compounds have shown significant glucosidase inhibition potential which can be used to treat type 2 diabetes mellitus. Cholinesterase inhibition potential has been used for the treatment of Alzheimer's disease (Rahman et al., 2021). Inflammation is a natural biochemical reaction of the body in response to infections and injuries caused by physical and chemical stimuli (Soulimani et al., 2021). The immune system responds to harmful stimuli by generating proinflammatory mediators, but moderate production of proinflammatory mediators leads to many lethal diseases. Phenolic compounds work in tandem with nonsteroidal anti‐inflammatory drugs (NSAIDs) to inhibit the gene expression or activity of proinflammatory mediators, including cyclooxygenase (COX). Phenolic compounds inhibit the excessive production of proinflammatory cytokine which leads to asthma and cancer (Devi et al., 2015). Phenolic compounds have also been demonstrated to treat skin diseases due to their anti‐inflammatory properties (Działo et al., 2016), rheumatoid arthritis (Nguyen et al., 2019), and inflammatory bowel disease (Singh & Easwari, 2021).

Phenolic compounds have the ability to treat enzyme‐linked human diseases such as neurodegenerative diseases and hypertension. The angiotensin‐converting enzyme (ACE) inhibition by phenolic compounds has been used to treat hypertension. Patten et al. reported the angiotensin‐converting enzyme (ACE) inhibition effect on 74 plant families (Patten et al., 2016). Polyphenols isolated from cocoa showed remarkable antihypertensive properties via ACE inhibition (Sari et al., 2020).

#### 4.1.2 Flavonoids

Flavonoids are widely distributed polyphenols mainly present in plant‐based foods and beverages. These are generally found in grapes, cherries, berries, plums, and crane berries and more than 8000 flavonoids have been identified. Two main categories of flavonoids are anthocyanins and anthoxanthins. Anthocyanins are responsible for plant and fruit color while anthoxanthins are colorless molecules that are further classified as flavones, isoflavones, flavanols, and flavanones. Anthocyanins, kaempferol, hesperidin, naringenin, myricetin, and quercetin are the chief flavonoids that have particular impact on human health due to their biological activities. Flavonoids possess anticancer activity and preventive potential against cell destruction due to oxidation processes. These compounds have been reported as immunomodulatory agents and have beneficial effect on the immune system (Khairan et al., 2021).

Flavanols, particularly monomeric flavanols, including catechin, gallocatechin, epigallocatechin, epicatechin, and proanthocyanidin (polymerization product of flavanols), are found in significant concentration in chocolate and cocoa powder, grapes, and teas. Thearubigin and theaflavin are present in significant concentrations in black tea. The most readily absorbable flavonoids are catechins because of their existence as unbound form and the only flavonoid not bound to sugar. Catechin and epicatechin which are found in high concentration in grape and grape juices are capable of reducing glutamate excitotoxicity and exerting powerful antioxidant activity by reducing the oxidation of low‐density lipoprotein (LDL) and platelet aggregation and ameliorating endothelial function (Mecocci et al., 2014). Among flavonols, quercetin along with myricetin and kaempferol is probably the most abundant flavonol in capers, green teas, apples, and onions with promising antioxidant potential. Quercetin has been considered beneficial for the treatment of neurodegenerative diseases such as Alzheimer's disease (AD) due to its impact on multiple action mechanisms related to these disorders. Isoflavones, such as genistein, puerarin, glycitin, and daidzein, present in increased concentration in soybeans have beneficial activity to prevent cognitive decline and maintain normal brain function which is still debatable. Luteolin, a flavone from the class of flavonoids, found in rosemary, celery, and parsley has demonstrated activity against neurodegenerative diseases (Bonetti et al., 2017).

#### 4.1.3 Lignans and stilbenes

Lignans belong to the wide class of phenolic compounds found in higher plants and are derived from dibenzylbutane as well as produced in vivo from human gut microorganisms. These are phytoestrogens present in legumes, cereals, vegetables, and fruits as glycosides and bio‐oligomers aglycones. The richest sources of lignans are sesame seeds, flaxseed, and linseeds. The most common lignans are syringaresinol, pinoresinol, sesamolin, sesamol, sesamin, matairesinol, secoisolariciresinol, 7‐hydroxymatairesinol, and lariciresinol. Lignans have antifeedant, antiviral, antioxidants, and insecticidal properties (Banwo et al., 2021).

Resveratrol is the most widely studied stilbene known to have cardioprotective and anticarcinogenic activities. Resveratrol is found in berries, grapes, vine, and peanuts. The cardioprotective function of resveratrol stilbene is achieved by the prevention of vascular smooth muscle cell proliferation (Delgado et al., 2019).

### 4.2 Alkaloids

Plant alkaloids are one of the largest groups of natural entities, representing a diverse group of chemical products. The term alkaloid was introduced for the first time by W. Meisner in the early 19th century to name the natural substances behaving like bases. There is no precise definition of alkaloids, and it is difficult to differentiate alkaloids from other natural metabolites containing nitrogen as an essential component (Fatima et al., 2020). Alkaloids are defined as nitrogen‐containing nonnucleosidic and nonpeptidic compounds. Alkaloids are organic bases that have nitrogen‐containing heterocyclic rings and majority of them possess definite pharmacological activities (Bari et al., 2012).

Alkaloids have a 3000 years golden history in human medicine used as laxative, astringent, and sedatives for snake bites, fever, and insanity. About 5500 alkaloids are known, comprising the largest class of phytobioactive compounds, and are widely distributed (Mehmood et al., 2012). They are known to have therapeutic potential and are used as medicational and recreational drugs. Most of the alkaloids are very toxic and bitter in taste which makes them useful for plants to be used as defensive agents against invertebrate pest attacks, microbial pathogens, and herbivores (Khadka et al., 2021). Several medicinal plants containing alkaloids have been manipulated by human beings as pain relievers (Iqbal et al., 2013).

Alkaloids form salts with organic acids and mineral acids. Alkaloid salts are usually soluble in dilute alcohols and water while rarely insoluble in organic solvents. They are classified according to their chemical nature and natural sources. Alkaloids are commonly classified according to the distribution of their C–N skeleton into the following large groups, such as diterpenoid, steroidal, quinazoline, indole, isoquinoline, quinoline, pyridine, pyrrolidine, and other alkaloids (Hafiz et al., 2018).

Cytotoxicity, carcinogenic or mutagenic activity, antifungal, antiviral, and antibacterial activities, and their possible roles as phytoalexins have been reported. Many alkaloids are highly toxic and may lead to the death of animals if eaten. Several alkaloids such as anabasine and nicotine are used as insecticides (Jahangir et al., 2018). Plants containing berberine alkaloids are known to be used as antiseptics, analgesics, antistomatitis, and sedatives in Chinese folk medicine. In Islamic and Indian folk medicine, such plants are used for eye diseases and bleeding disorders, and as uterine muscle depressants, sedatives, and antiseptics. Both quaternary and their tetra‐alkaloids derivatives possess several biological and therapeutic properties, for example, tetrahydropalmatine, jatrorrhizine, and palmatine have been reported for their in vitro antimalarial activity (Adhikari et al., 2021). In China, tetrahydropalmatine has been tabulated to exhibit sedative, hypotensive, and bradycardial activities and is used as an analgesic. About 40% of modern drugs are derived from natural sources and a wider pharmacological potential is exhibited by alkaloids, especially isoquinoline (Afzal et al., 2018).

Alkaloids have remarkable ability to inhibit animal and human coronaviruses (Khan et al., 2018). Songorine (C20 diterpenoid alkaloid) and its derivatives possess several therapeutic effects including anti‐inflammatory, antiarrhythmic, anticardiac fibrillation, anxiolytic effects, excitation of synaptic transmission, antinociceptive and antiarthritic effects (Fielding et al., 2020). Neurodegenerative diseases are characterized by neuron deterioration which is affecting millions of people worldwide. Currently, pharmaceutical approaches are unable to cure or even halt the neurodegenerative disease progression. For the last two decades, much attention has been paid to the antineurodegenerative and neuroprotective properties of natural compounds with high bioavailability. Several types of research have reported the antineurodegenerative properties of berberine, an isoquinoline alkaloid isolated from medicinal herbs. Berberine has the inhibitory potential against numerous pathogenic enzymes, triggers autophagy, attenuates neuroinflammation, and protects neurons against apoptotic cell death (Fan et al., 2019). Berberine has also shown promising therapeutic potential, including anticancer, anti‐inflammatory, analgesic, antioxidant, cardioprotective, antihyperlipidemic, antidiabetic, antimicrobial, antinociceptive, antidepressant, memory enhancement, and cholesterol‐lowering effects. It also has the ability to lower lipid–glucose levels, hence can be used as antiatherosclerotic and anti‐Alzheimer's disease (Singh et al., 2019).

### 4.3 Terpenes

Terpenes, also called terpenoids or isoprenoids, constitute the largest class of natural products with over 55,000 known compounds. These chemicals constitute the secondary metabolism of animal and vegetal species resulting from C5 isoprene units connected in a head‐to‐tail fashion via the intermediates mevalonic acid. Typical structures containing carbon skeletons are classified as tetraterpenes (C40), triterpenes (C30), sesterterpenes (C25), diterpenes (C20), sesquiterpenes (C15), monoterpenes (C10), and hemiterpenes (C5; Noreen, Zia, et al., 2020).

Terpenes are widely applied in the industrial sector as spices, fragrances, and flavors and are used in scents, cosmetic products, and as food additives. They are active components of drugs in pharmaceutical industry (Shahid et al., 2020). Increasing interest in the therapeutic application of alkaloids among these pharmaceuticals, the antimalarial (artemisinin) and anticancer (paclitaxel) drugs are two of the well‐known terpene‐based drugs (Ashraf et al., 2020).

Monoterpenes in essential oils have the ability to damage microbial cells and inhibit microbial growth (Aslam et al., 2011). Terpenes have remarkable anti‐inflammatory potential, it perform anti‐inflammatory activity by inhibiting inflammatory pathways associated with numerous diseases (Riaz et al., 2017). Terpenes isolated from turmeric species (*Curcuma* sp.) showed anti‐inflammatory properties, both in vitro and in vivo analyses (Tanweer et al., 2018). Terpenes have enzyme inhibition activities as they have potential to inhibit lipoxygenase and proteinase, principal enzymes of inflammatory pathways (Irshad et al., 2018). Terpenes inhibit oxidative stress by inhibiting the catalytic activity of enzymes involved in reactive oxygen species generation and by forming metal‐ion chelates. Camphor is a monoterpene widely used in cosmetics, pharmaceutics, and the food industry (Bhatti et al., 2013).

**FIGURE 5:**
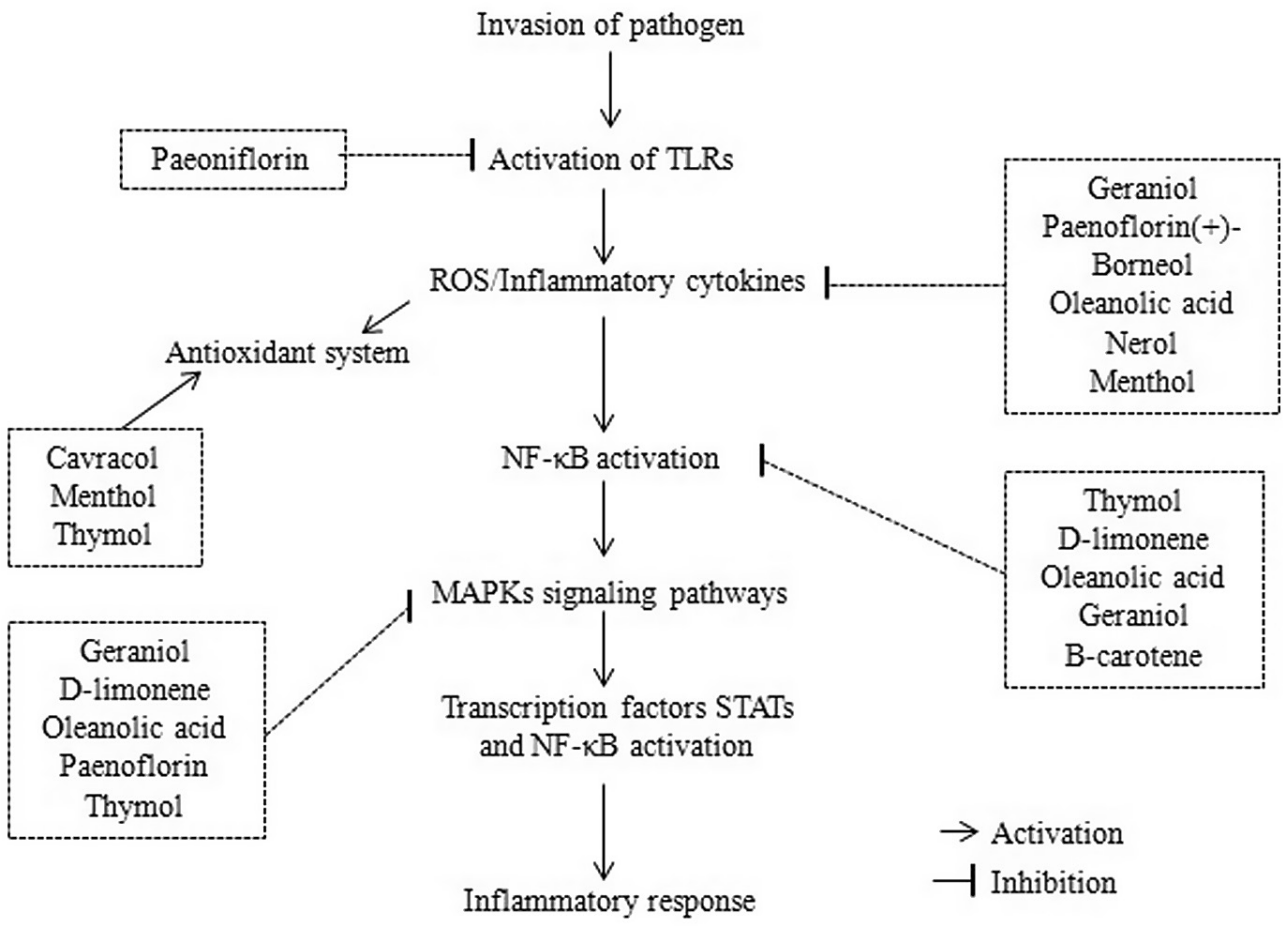
Possible molecular mechanism of anti‐inflammatory activity of plant terpenes.

Terpenoids inhibit apoptosis of hepatic cells through the inhibition of cytosolic release of cytochrome c, reducing the Bax/Bcl‐2 ratio, and by the inhibition of I‐κB, ERK, and JNK phosphorylation. They are also involved in the management of disorders caused by obesity, such as insulin resistance, type‐2 diabetes, and hyperlipidemia (Chatterjee et al., 2019). Antioxidant properties of terpenoids are also associated with their hepatoprotective activity (Ielciu et al., 2021). Terpenoids are supposed to be a safe and promising agent for the treatment of diabetes (Panigrahy et al., 2021). The antimalarial activity of terpenoids demonstrated a similar action mechanism to that of chloroquine (a pharmaceutical drug). Curcumin is widely used for the treatment of various diseases such as anti‐inflammatory, antioxidant, anticancer, antiplasmodial, antiseptic, and anticancer (Cox‐Georgian et al., 2019). A schematic molecular mechanism of the anti‐inflammatory activity of terpenes is shown in Figure 5.

### 4.4 Saponins

Saponins are known as surface‐active compounds and are widely distributed in the plant kingdom. Saponins include five‐ring triterpene saponins and four‐ring triterpene saponins which are reported to have cardioprotective potential through diverse mechanisms involving calcium homeostasis, regulation of energy metabolism, and the inhibition of inflammation and oxidative stress (Chen et al., 2017). A steroidal saponin called polyphyllin I (PPI) is extracted from *Paris polyphylla* roots. PPI is anticancer and cardioprotective. Its anticancer activity is through the inhibition of tumor cell growth and proliferation. It activates NF‐κB and reduces oxidative stress and inflammation resulting in decreased myocardial death (Huang et al., 2020). Platycodin D is another saponin found in *Platycodon grandiflorus*, which has antioxidant potential and anti‐inflammatory and antiatherosclerotic activities (Wang, Che, et al., 2018). Gypenoside A from *Gynostemma pentaphyllum* possesses anti‐inflammatory, antioxidative, and antitumor activities (Chang et al., 2020). Ginsenoside Rb3 is a saponin mainly found in *Panax ginseng* C. A. Meyer plant which is antiapoptotic inhibiting apoptosis through the activation of NK/NF‐κB signaling pathway (Ma et al., 2014).

### 4.5 Polysaccharides

**FIGURE 6:**
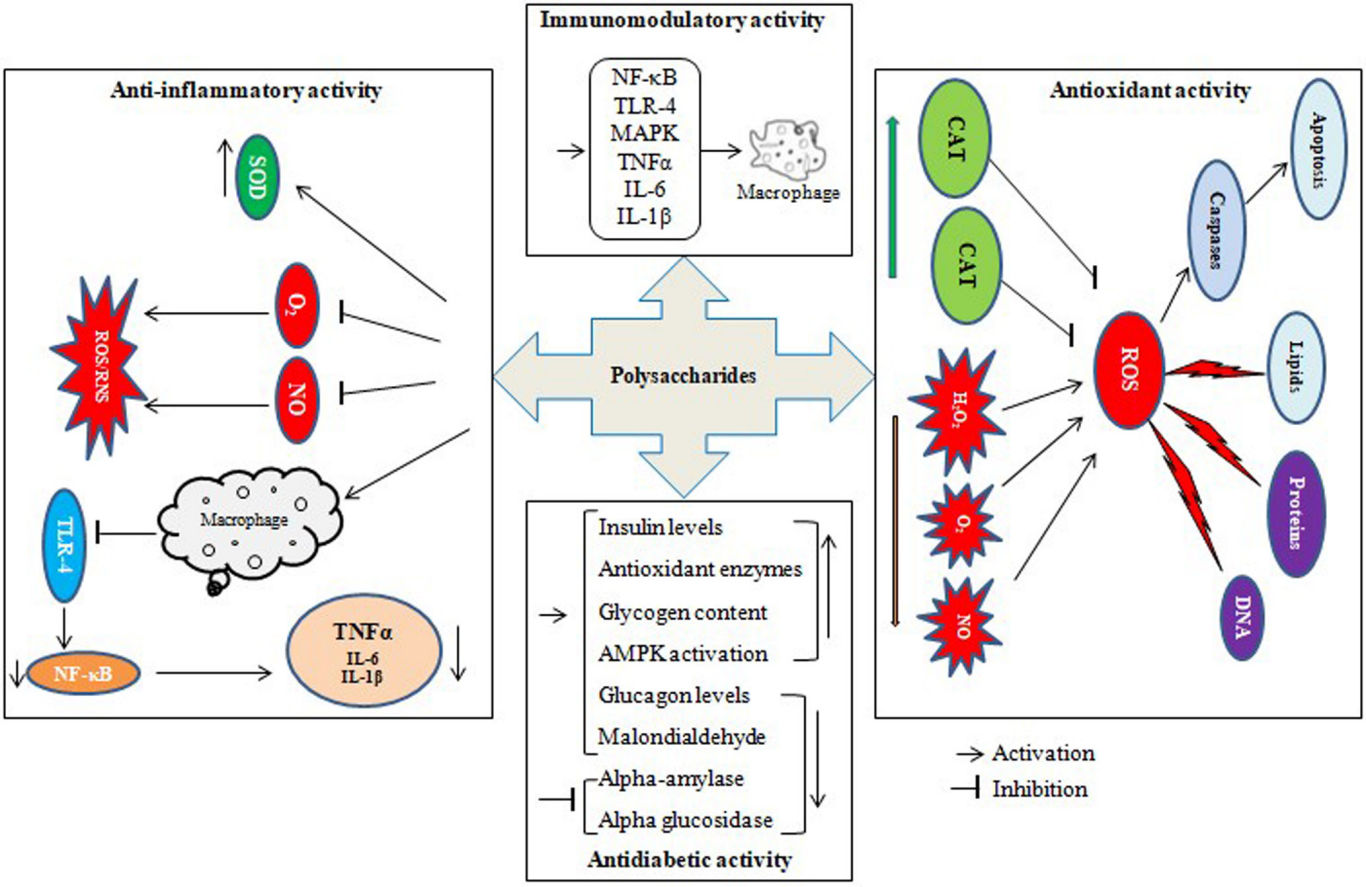
Possible action mechanism of plant polysaccharides as antioxidant, anti‐inflammatory, antidiabetic, and immunomodulatory agents. Plant polysaccharides increase the endogenously produced antioxidant enzymes like catalase and superoxide dismutase levels, whereas significantly reduce ROS‐induced free radicals, which are responsible for the destruction of important biomolecules found in body tissues that lead to apoptosis. These polysaccharides are primarily involved in the downregulation of toll‐like receptor 4 (TLR‐4) and NF‐κB, a nuclear transcription factor involved in inflammatory diseases by triggering the inducible NO synthase, TNFα, IL‐6, and IL‐1β. Antidiabetic activity of plant polysaccharides has been shown through various mechanisms including antioxidative and anti‐inflammatory properties by alleviating β‐cell dysfunctioning, increasing insulin secretion, and inhibiting the α‐amylase and α‐glucosidase activity to improve glucose metabolism. Plant polysaccharides exhibit immune‐modulatory activity by inducing ROS and cell proliferation and increasing cytokines and macrophages phagocytosis.

Polysaccharides are generally found in vegetables and fruits. Several studies reported the cardioprotective potential of polysaccharides through various mechanisms like anticancer, antioxidative stress, anti‐inflammatory, immunomodulatory, and regulating metabolism. The biologically important polysaccharides are gums and fucoidan (Chen et al., 2021). The possible mechanism of polysaccharides action is shown in Figure 6.

Gums are polysaccharides obtained through extraction or natural exudation from different plant parts (Naqvi et al., 2011). Different monosaccharide units are linked together through glycosidic linkages forming the gums (Hussain, Ali, et al., 2015; Hussain, Bakalis, et al., 2015). On the basis of structure, composition, behavior, and production differences, gums have been classified into subcategories (Munir et al., 2015), broadly, exudates gums (synthesized by plants for protection against microbial attacks or in response to mechanical injury and process is named as gummosis) and nonexudate gums (artificially procured from plant by suitable extraction method). Gums have remarkable applications at commercial scale (Munir et al., 2016).

Plant gums possess several applications in food industry. Their use in food products to improve quality has been reported in the literature (Munir et al., 2017). However, in addition to numerous functionalities, gums have also been known for their health benefits (Ayub et al., 2018). Gums act as dietary fiber. Consumption of dietary fibers contributed to decreased risk of cardiac issues, promotes immunity and satiety, and contributes to the management of body weight (Irshad et al., 2011). Several studies have documented the role of gums as bioactive such as antioxidant and scavenging activity against free radicals which are necessary for decreasing oxidative stress (Ullah et al., 2019).

Guar gum like other natural gums is hydrophilic nontoxic, economical, and easily available. Being biodegradable has several applications in pharmaceutical, textile, and food industries. Due to biodegradation, it is less stable and cannot be used in its natural form. Surface modification via grafting enhances its uses as grafted gum is an important medium for controlled drug release (Shahid et al., 2013). Crude, purified, and hydrolyzed guar gum characterized through TGA, XRD, FTIR, and SEM demonstrated that hydrolyzed and grafted gums are more crystalline and increase thermal stability (Anjum et al., 2014; Bukhari et al., 2014).

## 5 EXTRACTION OF BIOACTIVE COMPOUNDS

Considering the variations among phytobioactive compounds and variety of plant species, it is essential to formulate a standard technique for the screening of phytobioactives from plants extract. Different extraction techniques have been used for the selective extraction of bioactive compounds from natural sources (Abbas et al., 2014). Most of the techniques used for bioactive extraction remain almost same through hundreds of years (Hussain, Ali, et al., 2015). All these assays have common objectives such as (a) extraction of target phytobioactives from plants extract, (b) enhancing the analytical bioassay selectivity, (c) enhancement of the sensitivity of bioassay, (d) conversion of phytobioactives into a more suitable form for detection and separation, and (e) the establishment of a strong and reproducible method that is independent of variations in the sample matrix (Iqbal et al., 2015).

Extraction techniques of phytobioactive compounds depend on the nature of the solvents used for extraction, mixing, and application of heat (Jabbar et al., 2012). Extraction techniques are classified into two major groups such as conventional extraction techniques (maceration, Soxhlet extraction, and hydrodistillation) and unconventional extraction techniques (Zia et al., 2012). New and promising techniques for phytobioactive compound extraction are introduced to overcome the limitations of conventional extraction methods. Some of the new and promising extraction techniques are microwave‐assisted extraction, enzyme‐assisted extraction, ultrasound‐assisted extraction, supercritical fluid extraction, pulsed electric field‐assisted extraction, and pressurized liquid extraction (Afzal, Shahid, Iqbal, & Hussain, 2014; Afzal, Shahid, & Jamil, 2014).

Microwave‐assisted extraction (MAE) uses electromagnetic waves that generate heat by penetrating inside the matrix resulting in subsequent cell wall destruction. The heat generated through microwave exposure causes cell wall swelling by inducing an increase in pressure and temperature of water vapor inside the cell, consequently releasing the intracellular compounds. Ultrasound‐assisted extraction (UAE) is capable of improving the transfer of heat and mass by rupturing the plant cell walls due to cavitation effect. Supercritical fluid extraction (SFE) works in two ways i‐e extraction and separation. In the first stage, fluid is brought to the required temperature by compressing to the required pressure and then diffuses into the sample to dissolve the soluble material and transfer the fluid into the next section for separation. In the separation stage, temperature and pressure are readjusted to reduce the solubility of supercritical fluid resulting in precipitation of the analytes (Carpentieri et al., 2021).

## 6 BIOASSAYS FOR PHYTOBIOACTIVE COMPOUNDS

After a plant has been identified as a medicinal plant, the bioactive compounds are detected by using so‐called “Bioassays” (Zahid et al., 2016). There are several procedures available for in vitro and in vivo screening of plant extract. In this process, successive extraction of medicinal plants is carried out using solvents of increasing polarity and tested by a range of bioassays according to the activity of bioactive molecules (Ali et al., 2016). When the bioactivity has been identified, combination of chromatographic methods has been performed for further separation (Rehman et al., 2013). After obtaining a pure compound, a variety of spectroscopic methods like MS, IR, and NMR are used for the structural elucidation, and X‐ray crystallography is used in certain cases (Riaz et al., 2017). After toxicity study, clinical trial is the last, but the most important part to determine and compare the efficacy and doses of the new medicinal product to the already available products in the market (Rubab et al., 2016).

### 6.1 Antioxidant assays

Antioxidants are molecules that have the ability to scavenge free radicals produced during oxidative stress (Ashraf et al., 2016). All organisms have natural defense systems that scavenge free radicals but present‐day lifestyle causes overproduction of free radicals which are responsible for oxidative stress. Oxidative stress imposed several deleterious effects such as damaging the cell structures, including proteins, lipids, and DNA (Hina et al., 2017). Many pharmaceutical drugs have been formulated for the treatment of oxidative stress (Iqbal et al., 2017). Synthetic antioxidants have a few limitations as they cause carcinogenicity and are expensive. Natural antioxidants (vitamin C, vitamin E, xanthophylls, tannins, carotenes, and phenolic) have several biological activities (Cheema et al., 2011), which is why scientists have been exploring natural antioxidants for use in medicine and food to replace the synthetic compounds (Riaz, Rasool, Bukhari, Shahid, Zubair, et al., 2012). Bioactive compounds of medicinal plants have antioxidant potential (Irshad et al., 2012) and are being explored by researchers extensively (Asghar et al., 2017). Different bioassays have been performed to explore the antioxidant properties of phytobioactives (Riaz et al., 2017). Some bioassays are as follows:

#### 6.1.1 DPPH assay

The radical scavenging DPPH bioassay (2, 2‐diphenyl‐1‐picrylhydrazine) was described by Blois in 1958 and was slightly modified by numerous researchers (Afzal, Shahid, Iqbal, & Hussain, 2014; Afzal, Shahid, & Jamil, 2014). DPPH assay is one of the most commonly used assays to determine the antioxidant activity of plant extract. The stable free radical DPPH reacts with hydrogen‐donating compounds. In its reduced form, DPPH is violet in color and turned into pale yellow color upon oxidation by an antioxidant. The activity of antioxidants is calculated by a reduction in optical density. In this method, DPPH solution (0.004%) in methanol is prepared and 10 μL of the sample was mixed with 1 mL of 0.004% DPPH solution (0.004%). Following incubation for 30 minutes, absorbance was measured at 517 nm (Irshad et al., 2017).

$$\text{DPPH Inhibition}\;\left(\%\right)=\left(A_{1}-A_{0}/A_{1}\right)\times 100$$

where *A*_1_ is the unknown sample absorbance and *A*_0_ is the blank absorbance.

#### 6.1.2 ABTS assay

The ABTS radical scavenging assay was developed by Rice Evans and Miller in 1994 and then modified by Re in 1999. The modification is based on the production of a radical cation through metmyoglobin activation with H_2_O_2_ in the presence of ABTS•+ (Anwar et al., 2013). This assay is extensively used, generating a blue/green ABTS•+ chromophore via ABTS and potassium persulfate reaction. ABTS radical scavenging method together with DPPH assay is one of the widely used assays for the determination of antioxidant potential of plant samples. The ABTS radical scavenging activity is measured at 734 nm with the help of a spectrophotometer. This bioassay measures the total antioxidant capacity in both hydrophilic and hydrophobic substances. Trolox, a water‐soluble analog of Vitamin E, is used as a positive control and the antioxidant activity is expressed as Trolox‐equivalent antioxidant capacity per mg of extract sample (TEAC/mg; Anjum et al., 2014; Sridhar & Charles, 2019).

#### 6.1.3 TPC assay

Folin–Ciocalteu method is used for the measurement of total phenolic content (TPC) based on phenolic group containing phytobioactives present in plant samples. The reaction for electron transfer occurs between antioxidants and molybdenum, reducing the complex‐forming blue species, which can be calculated by spectrophotometer. Sodium carbonate is added to make the medium alkaline which is required for this reaction, and gallic acid is used as standard (Waseem et al., 2021).

#### 6.1.4 TFC assay

Total flavonoid contents (TFC) in the plant extracts are determined by aluminum chloride colorimetric assay. The sample solution of plant extracts was mixed with 0.1 mL 10% aluminum nitrate, potassium acetate, and 80% ethanol. The reagents were allowed to stand for 40 min after thorough mixing and the absorbance was measured at 510 nm via spectrophotometer. The presence of flavonoids is indicated by the yellow coloration of the reaction mixture and quercetin is used as standard (Ayub et al., 2017).

#### 6.1.5 FRAP assay

Ferric ion‐reducing antioxidant power assay (FRAP) was developed by Iris Benzie and J. J. Strain and has been conducted in laboratories for the analysis of antioxidants due to its simplicity, good reproducibility, and simple instrumentation (Pramila & Julius, 2019). FRAP assay is based on the ability of antioxidants to reduce ferric ions in the presence of 2,4,6‐tris(2‐pyridyl)‐s‐triazine (TPTZ) at pH 3.6, forming an intense‐blue Fe^2+^ ‐TPTZ complex. The absorbance is measured at 593 nm. The absorbance is inversely proportional to the antioxidant content. For this assay, 0.05 mL of the extract is added to 1 mL of FRAP reagent in acetate buffer and the reaction mixture is incubated at 37°C for 30 min, and absorbance is measured at 593 nm. FRAP reagent is prepared by mixing 0.3 M acetate buffer (pH 3.6) and 10 mM 2,4,6‐tris(2‐pyridyl)‐S‐triazine in 40 mM hydrochloric acid and ferric chloride (20 mM) at a ratio of 10:1:1 (v/v/v; Saini et al., 2020).

#### 6.1.6 CUPRAC assay

Cupric reducing antioxidant capacity (CUPRAC) assay was developed by Apak, Guclu, Ozyurek, and Karademir (Özyürek et al., 2011) from the Department of Analytical, Istanbul University, 7 years following the development of the FRAP assay. This assay was developed mainly for the determination of “total antioxidant” as a nutritional index for food labeling. No doubt, CUPRAC assay has been proven to be an efficient method for a variety of polyphenols (namely phenolic acids including hydroxybenzoic acids and hydroxycinnamic acids, flavonoids, anthocyanins, and carotenoids), in addition to thiols, vitamin E, and vitamin C. Since its development, it has been used over the last few years by many investigators in different laboratories. The light‐blue chromogen Cu (Nc)2^2+^ used in this assay, upon reduction by the antioxidant, is reduced into an orange–yellow bis(neocuproine) copper (I) chelate Cu (Nc)2^+^. For this assay 100 μL of extract is added to the reaction mixture CuCl_2_ (200 μL, 10 mM), neocuproine (200 mL, 7.5 mM), and ammonium acetate aqueous buffer (200 μL, 1 M) at pH 7, incubated for 30 min at room temperature and measured the absorbance at 450 nm. CUPRAC capacity is expressed as Trolox, gallic acid, or quercetin equivalent (standard compound; Diniyah et al., 2020).

### 6.2 Antimicrobial activity

Antibiotics are used for the treatment of microbial infections and also as chemotherapeutic agents against infectious diseases (Hussain et al., 2010). However, few pathogens become resistant to antibiotics with the passage of time (Riaz et al., 2019). These drug‐resistant pathogens contribute to morbidity, mortality, and increased health issues (Mansoor et al., 2019). Resistance to antibiotics has become a global concern (Anwar et al., 2009). The therapeutic potential of several available antibiotics is being threatened by the appearance of multidrug‐resistant pathogens (Shahzadi et al., 2019). The use of crude plant extract and phytobioactive compounds are of huge significance to cope with multidrug‐resistant pathogens (Irshad et al., 2020).

Biofilm formation is a significant virulence strategy for microorganisms to survive in harsh environments (Iram et al., 2013). Bacteria form biofilms both on biotic and abiotic surfaces. Biofilms help bacteria in spreading infections by preventing them from antibiotics and the host immune system (Maqbool et al., 2019). Almost 80% of bacterial infections in humans are caused by biofilm. Biofilms cause numerous diseases, for example, vaginitis, gingivitis, conjunctivitis, colitis, and urethritis. *Pseudomonas aeruginosa* causes lung infection by forming a biofilm. *Staphylococcus aureus* and *Escherichia coli* also cause infections by forming biofilms. Biofilm formation on medical devices poses serious health issues. Plant extracts have been gaining huge attention to control emerging antibacterial resistance. Plants are packed up with bioactive compounds. Scientists have been evaluating various medicinal plants for their antibiofilm potential for the last few decades.

Quorum sensing (QS) is an intercellular communication process adopted by both Gram‐negative and Gram‐positive bacteria which is based on the secretion and detection of external signal molecules (Misbah Bhatti et al., 2021). In the form of biofilm, bacteria communicate with each other via quorum sensing. Quorum sensing is based on bacterial cell density. Inhibition of quorum sensing has the ability to inhibit virulence of bacteria and biofilms also (Afzal et al., 2021). Compounds having anti‐quorum‐sensing properties are able to control microbial infections, which is why researchers in the medical field are interested to explore more antimicrobial compounds (Abbasi et al., 2020). Phytobioactive compounds have remarkable antimicrobial properties (Shah et al., 2019). Lipophilic compounds isolated from *Moringa oleifera* bind with cytoplasmic membrane and inhibit the growth of filamentous fungi, especially by causing membrane permeabilization (Jabeen et al., 2008; Latif et al., 2011). Proteins purified from *Croton tiglium* L. showed remarkable antimicrobial activity (Shahid et al., 2008). Phytobioactive compounds isolated from sunflower seeds demonstrated high antibacterial properties (Den et al., 2019).

Broadly speaking, two types of bioassays are available for evaluating the antibacterial activity of natural compounds (Riaz, Rasool, Bukhari, Shahid, Zahoor, et al., 2012). Bioassays used for the detection of antimicrobial activity include diffusion‐based, bioautographic and cell morphology‐based assays. Quantitative bioassays are agar dilution, broth macrodilution, and broth microdilution assays (Abbasi et al., 2019).

### 6.3 Cytotoxicity analysis

When phytobioactive compounds are isolated as antioxidant and antimicrobial compounds, their toxicity analysis is quite necessary (Rasool et al., 2013; Sadaf et al., 2009). Most lethal poisons in the world, for example, botulinum, maitotoxin, batrachotoxin, and ricin, are naturally occurring compounds (Nighat et al., 2020; Shahid et al., 2020). In vitro bioassays for toxicity testing have increased considerably (Naseem et al., 2020). In vitro assays available for cytotoxicity testing are AMES assay (Jabbar et al., 2012; Rubab et al., 2015), DNA damage protection assay (Riaz et al., 2013), inflammatory assay (Ali et al., 2015), hemolytic assay (Gul et al., 2017; JaEeen et al., 2015; Shahid et al., 2016), thrombolytic assay (Rizwan et al., 2014; Sharif et al., 2018), etc. Several of the commonly used cytotoxicity assays are discussed below:

#### 6.3.1 AMES assay

AMES test has been used to evaluate the mutagenic potential of medicinal plant extract. It was standardized for the identification of mutagenic potential of chemicals in 1970s (Rasgele & Dulger, 2021). For the last few years, this test has been extensively used to assess herbal products. This mutagenic assay uses two mutant strains of *Salmonella typhimurium* (TA98 and TA100). Potassium dichromate (K_2_Cr_2_O_7_) is used as standard mutagen for TA98 and sodium azide (NaN_3_) for TA100. Pale‐yellow wells are considered positive wells. A number of positive wells in sample plate (plant extract) should be higher than the number of positive wells in the background plate for a sample to be mutagenic (da Silva Dantas et al., 2020).

#### 6.3.2 DNA damage protection assay

Oxidative stress is the main cause of aging and DNA damage. Nowadays, chemical, biological, and physical exposure and aging can increase DNA damage. DNA damage protection assay is used to assess the protective effects of plant extracts. CT‐DNA (calf thymus) is commonly used to determine DNA damage protection potential of extracts. Fenton's reagent is used to generate oxidative stress (Anjum et al., 2020).

#### 6.3.3 Anti‐inflammatory assay

Inflammation is the response of the immune system to injury and infection. Denaturation of proteins is a well‐documented cause of inflammation. Several phytobioactives possess the ability to inhibit protein denaturation. The in vitro anti‐inflammatory potential of plant extracts is evaluated by the BSA denaturation method. Bovine serum albumin (BSA) solution is prepared in dH_2_O. In test tubes, 0.45 mL BSA and 0.05 mL of test samples are poured and incubated at 37°C for 20 min after that the temperature is increased to keep samples at 57°C for 3 min. Diclofenac sodium is used as standard. On cooling at room temperature, 2.5 mL of PBS (phosphate buffer saline) is added to test tubes, and absorbance is measured at 255 nm. The anti‐inflammatory potential of plant extracts is compared with the positive control (diclofenac sodium; Yadav & Mohite, 2020).

Inhibition%=100×ODsample/ODcontrol−1

#### 6.3.4 Antihemolytic assay

The antihemolytic assay is used to evaluate the protective capacity of plant extracts for red blood cells. In this assay, fresh heparinized human blood is used. After removing the serum, the mean packed volume is washed with PBS solution. Blood suspension (180 μL) and plant extract (20 μL) are added in Eppendorf tubes, incubated for 30 min, and centrifuged for 5 min. After centrifugation, 100 μL supernatant from each Eppendorf tube is taken and diluted with 900 μL PBS solution. Subsequently, 100 μL from new Eppendorf tubes is poured into microwells in ELISA plates, and absorbance is measured at 576 nm by an ELISA reader. Triton X‐100 is taken as the positive control while PBS solution is taken as the negative control. The test was executed in triplicate (Kauser et al., 2018).

%Hemolysis=ODsample/ODcontrol×100

## 7 CHARACTERIZATION OF PHYTOBIOACTIVE COMPOUNDS

Phytobioactive compounds are separated and purified from plant extracts employing chromatographic techniques (Qazi et al., 2021). The most common chromatographic methods used are thin‐layer chromatography (TLC) and high‐performance liquid chromatography (HPLC; Alim et al., 2010; Naseem et al., 2020). Gas chromatography (GC) is another technique used for separating, identifying, and quantifying phytobioactive compounds such as flavonoids, phenolic acids, and condensed tannins (Sajid et al., 2016). GC technique is used for the analysis of volatile compounds only (Qasim et al., 2020). FTIR and NMR are used to determine the functional groups of compounds present in the crude plant sample and to detect the number of protons present in the compound.

### 7.1 Thin‐layer chromatography (TLC)

TLC is the most commonly used adsorption chromatographic technique, employed for the separation and analysis of low‐molecular‐weight phytochemicals where plant extract is separated based on the nature of stationary phase (Ullah, 2020).

### 7.2 High‐performance liquid chromatography (HPLC)

HPLC is an analytical technique for the separation and identification of organic and inorganic solutes in a variety of samples including pharmaceutical, biological, industrial, and environmental samples. HPLC separation of compounds is based on the interaction of compounds with solid particles of tightly packed columns (stationary phase) and the mobile‐phase solvent (Grybinik & Bosakova, 2021). HPLC is useful for samples that cannot be vaporized or decomposed at high temperatures. Nowadays, this technique is gaining popularity among other analytical techniques for the analysis of medicinal plant extracts (Sahu et al., 2018).

### 7.3 Gas chromatography (GC) and GC–MS

Gas chromatography is used for the analysis of volatile compounds which provides quantitative and qualitative information on individual components present in the compound such as essential oils, hydrocarbons, and solvents. Gas chromatography is based on the principle of adsorption and partition. In GC, sample is injected onto the top of the chromatographic column and then transported through the column (liquid stationary phase) by the flow of inert, gaseous mobile phase (Al‐Rubaye et al., 2017). GC–MS has become a highly recommended analytical technique in the pharmaceutical industry due to its robustness, selectivity, sensitivity, and reproducibility (Beale et al., 2018).

### 7.4 UV–Visible spectroscopy

UV–visible spectroscopy is used for the identification and qualitative analysis of biological mixtures and plant extracts. Phytobioactives can be analyzed by using UV–visible spectroscopy because aromatic compounds act as chromophores in the UV range. This technique provides information about the total phenolic contents of plant mixtures (Rocha et al., 2018).

### 7.5 Fourier transform infrared (FTIR) spectroscopy

FTIR spectroscopy is used for the identification of functional groups of the organic and inorganic compounds from plants extract by using infrared light. It helps in the identification, characterization, and structure determination of unknown molecules. It is a high‐resolution analytical technique for the identification and structural elucidation of phytobioactive compounds (Riaz et al., 2018).

### 7.6 Nuclear magnetic resonance (NMR) spectroscopy

NMR spectroscopy provides physical, chemical, and biological properties of matter. Due to its accuracy, rapidness, and intactness, NMR spectroscopy has gained a key role in determining the structures and dynamic properties of pharmaceutical drugs. Although NMR has remarkable advantages, sometimes it is combined with other techniques, such as HPLC, FT‐IR, and GC–MS, for more accurate and effective results (Cao et al., 2021).

## 8 CONCLUSIONS AND FUTURE PERSPECTIVES

Since ancient times, phytobioactive compounds are being used as traditional medicine for the treatment of several diseases around the globe. Several studies on their therapeutic potential are documented, as they are considered to be the natural sources for the production of new drugs with greater efficacy and biocompatibility. Recent publications are gradually adding up new characteristics to their pre‐existing vast spectrum pharmaceutical value. In present‐day lifestyle, oxidative stress has been continuously increasing and causing numerous disorders such as diabetes mellitus, cancer, and cardiovascular problems. Misuse of antibiotics results in antibiotic resistance which poses serious health issues, especially in hospitals. Synthetic drugs are insufficient to cope with these problems. Exploration of natural phytobioactive compounds to cure present‐day diseases is necessary. Polyphenols, alkaloids, and terpenoids have the potential to treat cancer, oxidative stress, inflammation, ulcers, diabetes, platelet aggregation, microbial resistance, and tumors.

This review study concluded that the use of bioactive compounds in foods, beverages, and pharmaceutical products plays a significant role in preventing various disorders including cancer, neurodegenerative diseases, oxidative stress, cardiovascular diseases, diabetes, obesity, etc. The antioxidant and other beneficial effects of phytobioactive compounds can be explained through the modulation of multiple pathways involved in cell signaling. These mechanisms of phytobioactive compound action include the modulation of caspases, NF‐kB, MAPK, and Nrf2 as well as LOX and COX. An important approach to prevent pathological processes is the possible crosstalk between these signaling pathways. One of the critical factors that can affect the potential of these phytobioactives to counteract the oxidative damage in human organs induced by ROS is bioavailability. The application of these phytobioactive compounds as therapeutic agents against human diseases will be facilitated through the understanding of their absorption, distribution, metabolism, and excretion. The complications encountered while using phytobioactives, like their stability, solubility, and bioavailability, need to be addressed to use these metabolites as medicine.

## ACKNOWLEDGEMENTS

The authors acknolwledge their resposective institutions for giving them time to work on this review.

## CONFLICT OF INTEREST STATEMENT

The authors declared no conflict of interest.

## Data Availability

Data sharing is not applicable—no new data are generated and this review article describes entirely the published literature.
